# Fine-mapping of *qGW4.05*, a major QTL for kernel weight and size in maize

**DOI:** 10.1186/s12870-016-0768-6

**Published:** 2016-04-12

**Authors:** Lin Chen, Yong-xiang Li, Chunhui Li, Xun Wu, Weiwei Qin, Xin Li, Fuchao Jiao, Xiaojing Zhang, Dengfeng Zhang, Yunsu Shi, Yanchun Song, Yu Li, Tianyu Wang

**Affiliations:** Institute of Crop Science, Chinese Academy of Agricultural Sciences, National Key Facility for Crop Gene Resources and Genetic Improvement (NFCRI), Beijing, 100081 China

**Keywords:** Maize, Kernel weight, Kernel size, Fine-mapping, Association mapping

## Abstract

**Background:**

Kernel weight and size are important components of grain yield in cereals. Although some information is available concerning the map positions of quantitative trait loci (QTL) for kernel weight and size in maize, little is known about the molecular mechanisms of these QTLs. *qGW4.05* is a major QTL that is associated with kernel weight and size in maize. We combined linkage analysis and association mapping to fine-map and identify candidate gene(s) at *qGW4.05.*

**Results:**

QTL *qGW4.05* was fine-mapped to a 279.6-kb interval in a segregating population derived from a cross of Huangzaosi with LV28. By combining the results of regional association mapping and linkage analysis, we identified GRMZM2G039934 as a candidate gene responsible for *qGW4.05*. Candidate gene-based association mapping was conducted using a panel of 184 inbred lines with variable kernel weights and kernel sizes. Six polymorphic sites in the gene GRMZM2G039934 were significantly associated with kernel weight and kernel size.

**Conclusion:**

The results of linkage analysis and association mapping revealed that GRMZM2G039934 is the most likely candidate gene for *qGW4.05.* These results will improve our understanding of the genetic architecture and molecular mechanisms underlying kernel development in maize.

**Electronic supplementary material:**

The online version of this article (doi:10.1186/s12870-016-0768-6) contains supplementary material, which is available to authorized users.

## Background

The corn kernel serves as a storage organ for assimilation products. Its yield directly influences food security. In agricultural production, maize yield is mainly composed of effective ear number, kernel number per ear and kernel weight. Kernel weight is the integrated embodiment of three elements: kernel length, kernel width and kernel thickness. Thus, understanding the genetic and molecular basis of kernel weight and kernel size is extremely important for the breeding of high-yield maize.

Due to the rapid development of molecular biotechnology, comparative genomics, and bioinformatics, many genes associated with maize flowering time, plant architecture and other traits, such as *vgt1* [1], *ZmCCT* [2, 3], *spi1* [4], *ZmCLA4* [5], *Fea2* [6, 7] and *tga1* [8], have been positionally cloned. However, genes directly related to kernel yield are rarely identified by natural genetic variation. Most genes associated with kernel yield are isolated by making use of maize mutants, such as *gln1-3*, *gln1-4, rgf1, sh1*, *sh2, dek1,* and *incw2* [9–13]. These genes identified by mutant analysis have facilitated the characterization of kernel development and its regulation. However, the genetic architecture and molecular mechanisms underlying natural quantitative variation in kernel yield have not been completely elucidated.

The genetic basis of quantitative traits can be recognized more clearly through QTL mapping. Many QTLs related to kernel traits have been identified in the maize genome [14–18], but few have been positionally cloned because 1) the maize genome is large and has many transposable elements and repetitive sequences [19–23] and 2) most complex traits such as kernel yield and kernel size are controlled by many genes with small effects [24–29]. QTLs identified in different genetic backgrounds across multiple environments have a higher chance of being positionally cloned. A QTL cluster on bin 4.05 of the maize genome has been repeatedly associated with kernel size and weight in different populations in previous studies. Doebley et al. (1994) identified a major QTL for kernel weight in BNL5.46 - UMC42A and UMC42A - UMC66 on bin 4.05 that explained 12.82 and 15.71 % of the phenotypic variance in two F_2_ populations developed from maize and teosinte, respectively [30]. Ajnone-Marsan P et al. (1995) identified a QTL associated with grain yield on bin 4.05 using the F_2_ population from a cross of B73 and A7 [31]. Peng et al. (2011) identified a QTL conferring kernel size and weight on bin 4.04–4.05 of the maize genome using two F_2:3_ populations [32]. These results demonstrate the importance of bin 4.05 for kernel size and weight and provide a target region for fine-mapping and positional cloning.

We previously identified a QTL cluster designated *qGW4.05* that is associated with kernel-related traits on bin 4.05 in the maize genome in different recombinant inbred line (RIL) populations across multiple environments [33]. The greatest effect of *qGW4.05* on kernel weight, kernel length and kernel width (23.94, 21.39 and 10.82 %, respectively) was observed in the RIL population of LV28 × HZS. These effects imply that this region carries a pleiotropic gene or several closely linked genes that affect both kernel size and weight. In this study, we used the excellent inbred line Huangzaosi (HZS) which plays an important role in Chinese maize breeding and has more than 70 inbred progeny lines and 80 important hybrids [34] and the RIL families from the cross of LV28 and HZS to develop a new mapping population. Then, we combined linkage analysis and regional association mapping to 1) re-evaluate the genetic effect of *qGW4.05* in the new population; 2) fine-map *qGW4.05*; and 3) infer potential candidate genes responsible for *qGW4.05.*

## Results

### Confirmation of *qGW4.05*

**Fig. 1:**
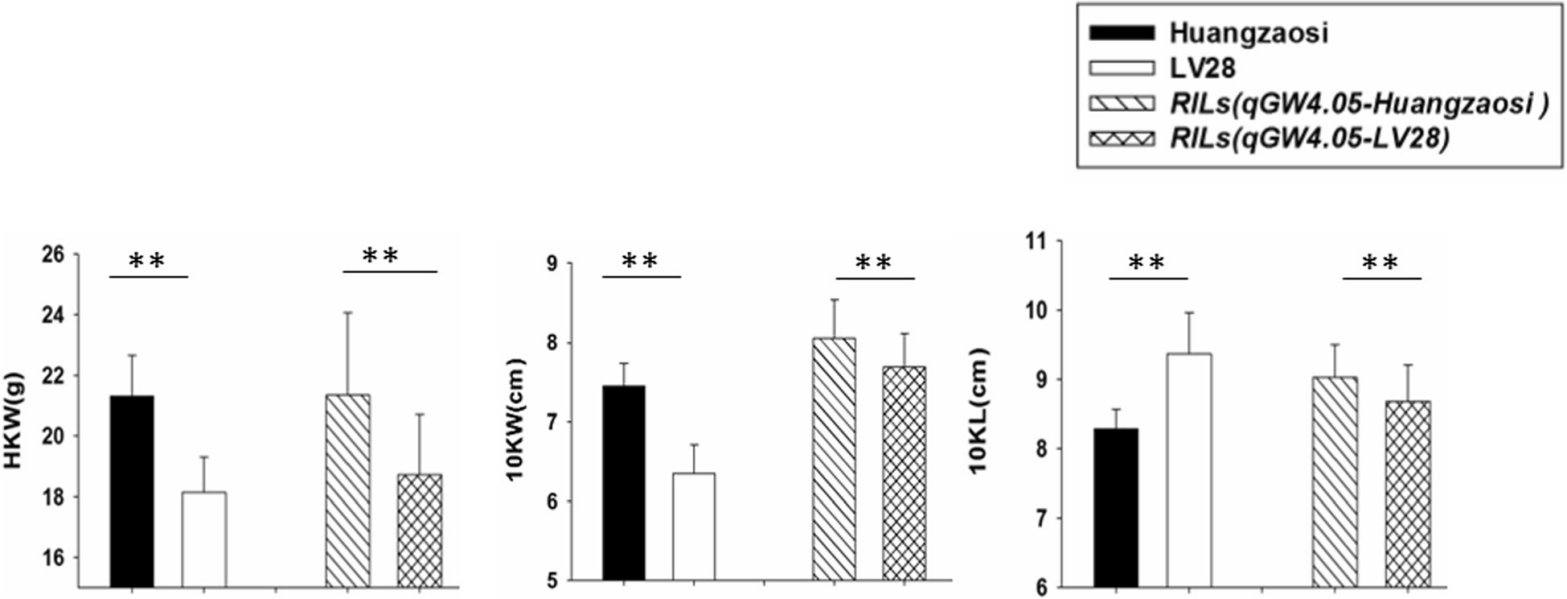
Phenotypic comparison among Huangzaosi, LV28 and the RIL families that harbour the Huangzaosi/LV28 allele on *qGW4.05*. HZS has a higher 100-kernel weight (21.30 g) than LV28 (18.10 g), a shorter 10-kernel length (8.20 cm) than LV28 (9.40 cm), and a wider 10-kernel width (7.40 cm) than LV28 (6.30 cm). The RIL families harbouring the *qGW4.05-HZS* allele have greater kernel weight and longer and wider kernels than those harbouring the *qGW4.05-LV28* allele (**P < 0.01)

**Fig. 2:**
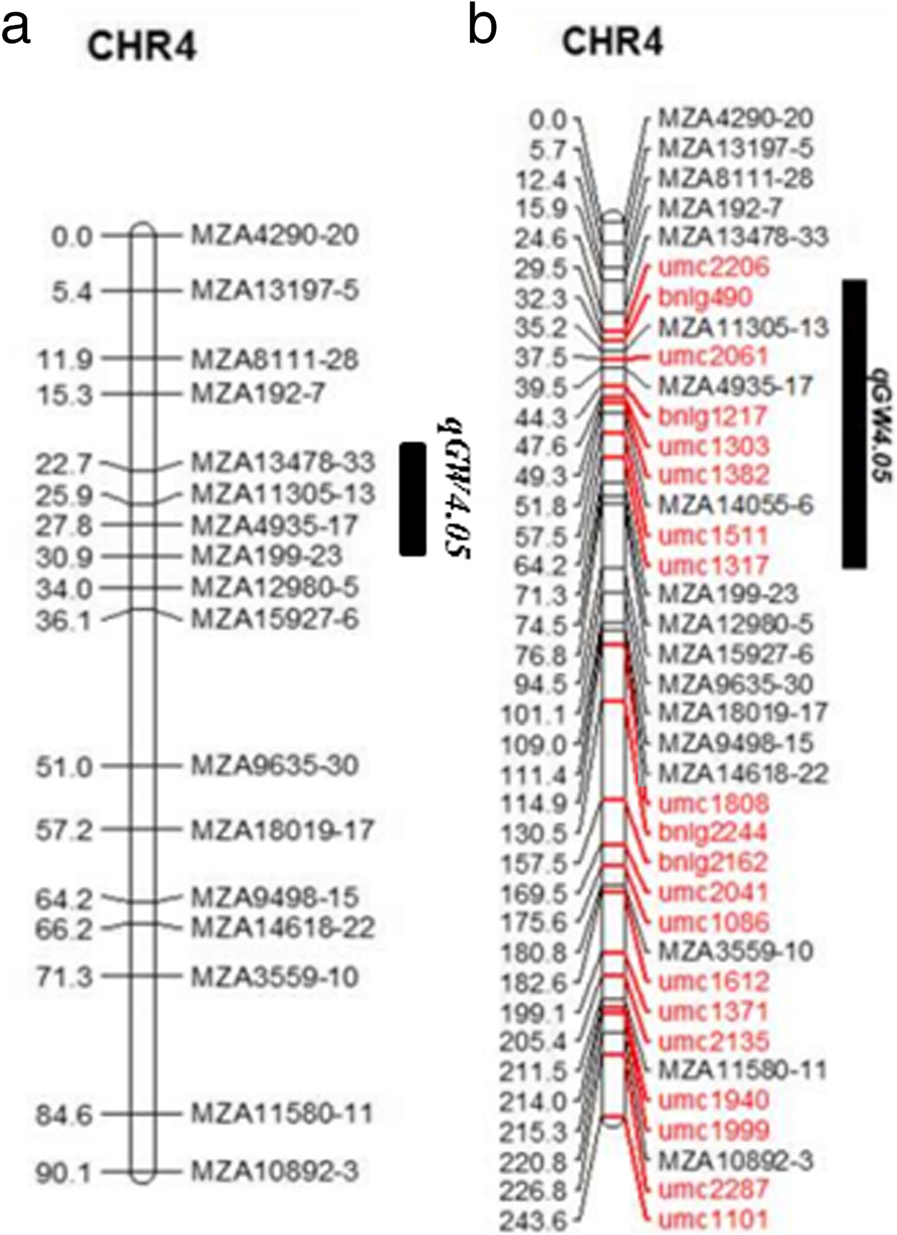
The location of qGW4.05 on the different genetic maps. **a** The genetic map constructed in 2013 and **b** the new genetic map constructed in this study. *qGW4.05* was located at MZA13478-33-MZA4935-17 in 2013, and it was re-mapped in the bnlg490-umc1511 region in this study

HZS and LV28 are elite inbred lines in Chinese maize breeding. HZS has a higher hundred kernel weight (21.30 g) than LV28 (18.10 g), a shorter 10-kernel length (8.20 cm) than LV28 (9.40 cm) and a wider 10-kernel width (7.40 cm) than LV28 (6.30 cm) (Fig. 1). To confirm the QTL on bin 4.05, we developed 20 new polymorphic markers (Additional file 1: Table S1) between LV28 and HZS on chromosome 4 and identified the genotype of all RIL families from LV28 × HZS. Subsequent re-mapping of *qGW4.05* to the interval bnlg490 - umc1511 on bin 4.05 explained 23.61, 20.52, and 10.0 % of the phenotypic variance in hundred kernel weight (HKW), 10-kernel length (10KL) and 10-kernel width (10KW), respectively (Fig. 2, Table 1). Using a flanking marker of *qGW4.05* to screen all RIL families, we determined that those RIL families harbouring the *qGW4.05*-HZS allele have greater kernel weight and longer and wider kernels than those harbouring the *qGW4.05*-LV28 allele (Fig. 1). This result is consistent with previous work [33] and indicates that *qGW4.05-HZS* plays a positive role in producing a larger kernel.

**Table 1:** QTLs detected in the different linkage map

Linkage map	Trait	Chromosome	Position (cM)^a^	Marker interval^b^	LOD^c^	PVE (%)^d^	Add^e^
Linkage map 2011	HKW	4	26	MZA11305-13 - MZA4935-17	10.86	23.94	−1.32
	10KL	4	25	MZA13478-33 - MZA11305-13	10.06	21.39	−0.24
	10KW	4	26	MZA11305-13 - MZA4935-17	5.08	10.82	−0.15
New linkage map 2012	HKW	4	36	MZA11305-13-umc2061	10.30	23.60	−1.31
	10KL	4	34	bnlg490-MZA11305-13	9.82	20.51	−0.23
	10KW	4	52	MZA14055-6-umc1511	4.78	9.97	−0.15

Notes: Position^a^, the genetic location of the QTL; Marker interval^b^, the flanking marker interval of the QTL; LOD^c^, Logarithm of odds for each QTL; PVE (%)^d^, percentage of phenotypic variance explained by a QTL; A^e^, additive values (a positive value indicates that the additive effect was derived from LV28, and a negative value indicates derivation from Huangzaosi)

Subsequently, we crossed the RIL family of G184, which harbours the *qGW4.05* allele from LV28, with HZS to produce an RIL-F_2_ population. Using these 1333 F_2_ plants in 2012, *qGW4.05* was mapped to the UMC2061-BNLG1217 interval (Additional file 1: Table S1). The allele of HZS displays partial dominance over the allele of LV28. The locus *qGW4.05* explained 5.17, 3.01, and 2.98 % of the phenotypic variance in kernel length, kernel width and kernel weight, respectively (Additional file 2: Table S2). These results confirmed that the UMC2061-BNLG1217 interval contains a functional unit controlling kernel size and weight in maize.

### Fine-mapping of *qGW4.05*

**Fig. 3:**
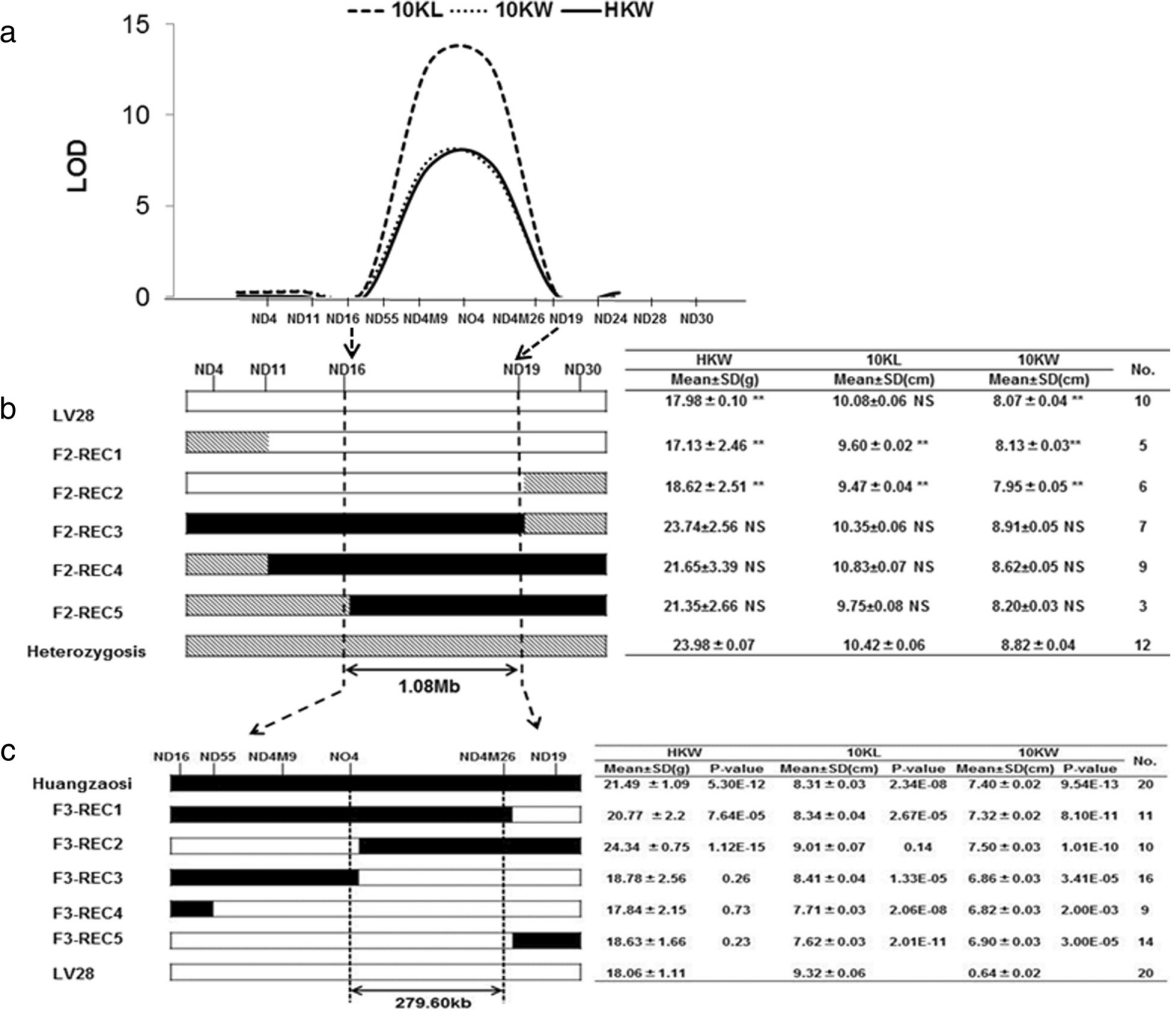
The process of map-based cloning of *qGW4.05*. **a** Location of qGW4.05 on chromosome 4, mapped using the F2 population in 2012. **b** and **c** The genotypes and phenotypes of different recombination types selected from the F2 population in 2012 and 2013. These recombinants of the two F2 populations were both classified into seven types. The genetic structure for each type is depicted as *black*, *white*, or *grey rectangles*, representing homozygous Huangzaosi/Huangzaosi, homozygous LV28/LV28, and heterozygous Huangzaosi/LV28, respectively. The tables on the *right* show the variations in 100-kernel weight, 10-kernel length and 10-kernel width of each recombinant type between different genotypes, and the total number (NO.) of plants refers to all plants of a given recombinant type in the F2 populations. **, significantly different at *P* < 0.01; *NS* no significant difference at the *P* < 0.01 level. These findings suggested that the *qGW4.05* allele from Huangzaosi can increase the 100-kernel weight, 10-kernel length and 10-kernel width. The interval of *qGW4.05* could be narrowed down from an ~1.08-Mb to an ~279.60-Kb region that was flanked by the markers NO4 and ND4M26

To improve the accuracy of the fine-mapping, we developed Indel (insertion and deletion) markers to replace the initial simple sequence repeat (SSR) markers; the initial SSR markers have a fuzzy physical location around *qGW4.05* on chromosome 4 (30–40 Mb) of the maize genome (Additional file 1: Table S1). Using the new Indel markers to genotype the RIL-F_2_ population, *qGW4.05* was further mapped to the ND16-ND19 interval by QTL analysis (Fig. 3a). *qGW4.05* explained 7.70, 8.88, and 7.34 % of the phenotypic variance in kernel length, kernel width and kernel weight, respectively, according to the results of the re-analysis (Table 2). This result is consistent with the QTL mapping using the initial SSR markers, indicating that the physical locations of these markers are the same. We then identified five recombinant types using the new markers on the 1332 F_2_ individuals in 2012, among which F2-Rec1 to F2-Rec2 carried the LV28 allele in the ND16-ND19 interval, whereas F2-Rec3 to F2-Rec5 carried the HZS allele in the corresponding interval (Fig. 3b). The 100-kernel weight of F2-Rec1 to F2-Rec2 was distinctly less than that of heterozygotes in this region and less than that of F2-Rec3 to F2-Rec5 (Fig. 3b), indicating that the ND16-ND19 interval may contain a QTL for kernel weight. Similar performance in kernel length and kernel width was observed (Fig. 3b), suggesting that the ND16-ND19 interval might contain a pleiotropic QTL.

**Table 2:** *qGW4.05* location in the F2 population in 2012

Trait	Chromosome	Marker interval^a^	LOD^b^	PVE (%)^c^	Add^d^	Dom^e^
10KL	4	ND16-ND19	8.63	7.70	−0.03	0.01
10KW	4	ND16-ND19	9.93	8.88	−0.03	0.01
HKW	4	ND16-ND19	8.12	7.33	−2.30	0.48

Notes: Marker interval^a^, the flanking marker interval of the QTL; LOD^b^, Logarithm of odds for each QTL; PVE (%)^c^, percentage of phenotypic variance explained by a QTL; A^d^, additive values (a positive value indicates that the additive effect was derived from LV28, and a negative value indicates derivation from Huangzaosi); D^e^, dominant values

A larger segregating population with 8000 F_3_ individuals was developed from the F_2_ plants, which are heterozygous in the ND16-ND19 interval, and used to fine-map *qGW4.05* in summer 2013. Furthermore, new markers were developed to identify recombinants in the ND16-ND19 interval. Using the same analytical method, we successfully narrowed *qGW4.05* to the NO4-ND4M26 interval in the maize genome, which is 279.6 kb long (Fig. 3c). There was no significant difference in kernel weight between LV28 and F3-Rec3 to F3-Rec5 carrying the LV28 allele in the NO4-ND4M26 interval on the maize genome (Fig. 3c). In addition, the kernel weight of F3-Rec1 to F3-Rec2 carrying the HZS allele in the NO4-ND4M26 interval was greater than that of LV28 (Fig. 3c). The kernel width of F3-Rec1 to F3-Rec2 was greater than that of LV28, and F3-Rec3 to F3-Rec5 carrying the LV28 allele in the interval were closer to LV28 than were F3-Rec1 and F3-Rec2 carrying the HZS allele of *qGW4.05*. However, the kernel length was the same between F3-Rec1 to F3-Rec3 and F3-Rec4 to F3-Rec5 (Fig. 3c). The unexpected kernel size performance can be attributed to the strong environmental influence on kernel-related traits. In conclusion, we confirmed that there is a gene controlling kernel weight that also likely affects kernel length and kernel width in specific environments.

### Validation of *qGW4.05* in the RIL population

**Fig. 4:**
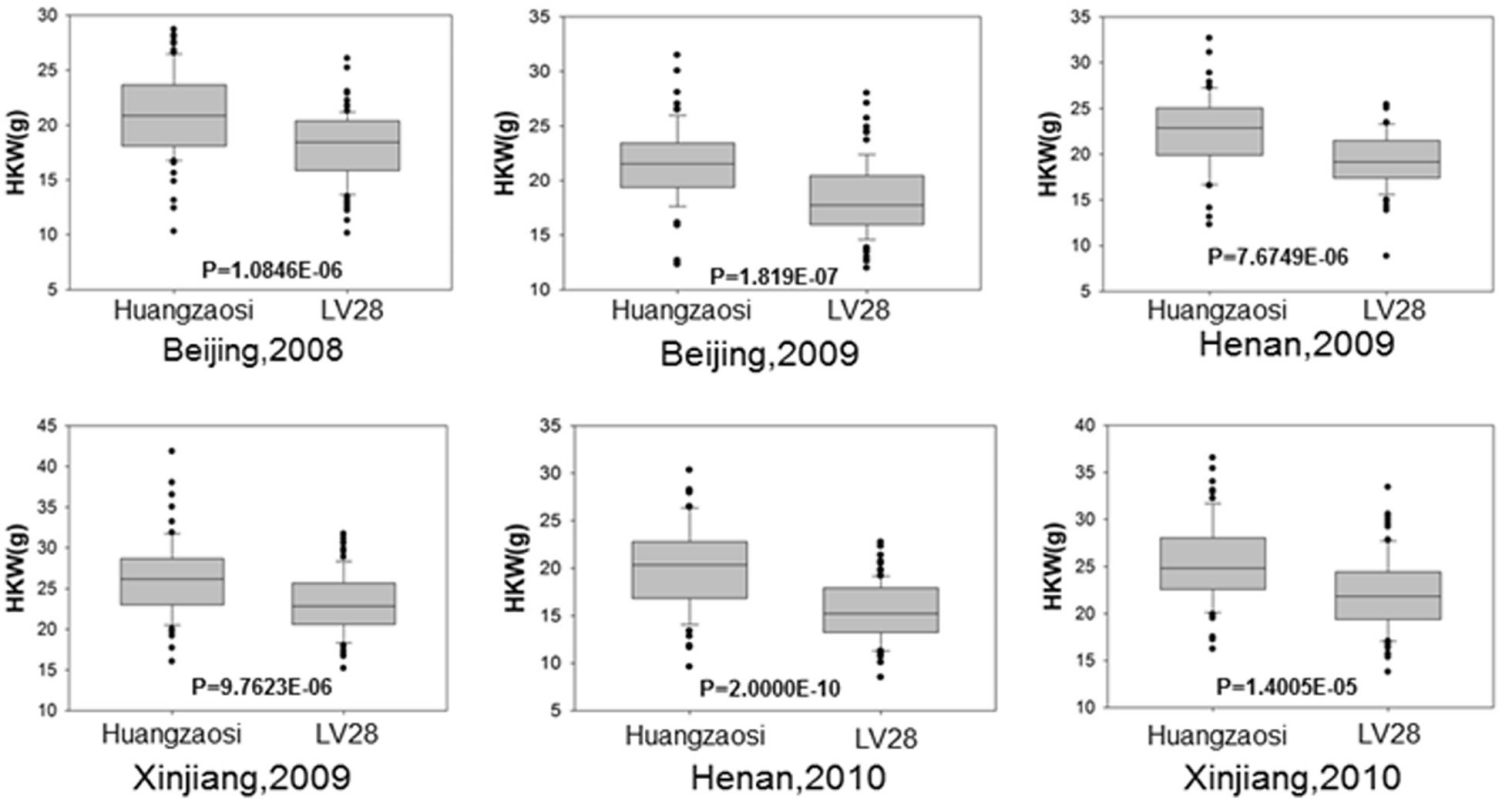
Validation of *qGW4.05* for hundred kernel weight (HKW) in the RIL population in six different environments. The RILs were genotyped by using the markers NO4 and ND4M26. The distributions and mean values for HKW are shown for the two homozygous genotypes, Huangzaosi and LV28, at six experimental sites. Compared with the RIL families with the LV28 homozygous genotype at the qGW4.05 region, the RIL families with the Huangzaosi homozygous genotype at the qGW4.05 region had significantly higher (*P* < 0.01) hundred-kernel weight across the six different environments

We next determined whether the restricted interval (NO4-ND4M26) is present in the RIL population from the cross of HZS and LV28 and has significant genetic effects on phenotypes. Kernel weight and kernel size were evaluated in six different environments [33]. We used the markers NO4 and ND4M26 to genotype the RIL population. Among the RILs, 68 and 79 families were homozygous for HZS and LV28, respectively. Kernel weight and kernel width differed significantly (*P* < 0.01) between the RILs homozygous for HZS and LV28 in all six environments (Fig. 4, Additional file 3: Figure S1), and kernel length differed significantly (*P* < 0.01) in all but the Xinjiang 2010 environment (Additional file 4: Figure S2). These findings suggest that the QTL in the interval of NO4-ND4M26 can affect kernel weight and kernel size in the RIL population, which is in agreement with our previous fine-mapping results.

### Regional association mapping

**Fig. 5:**
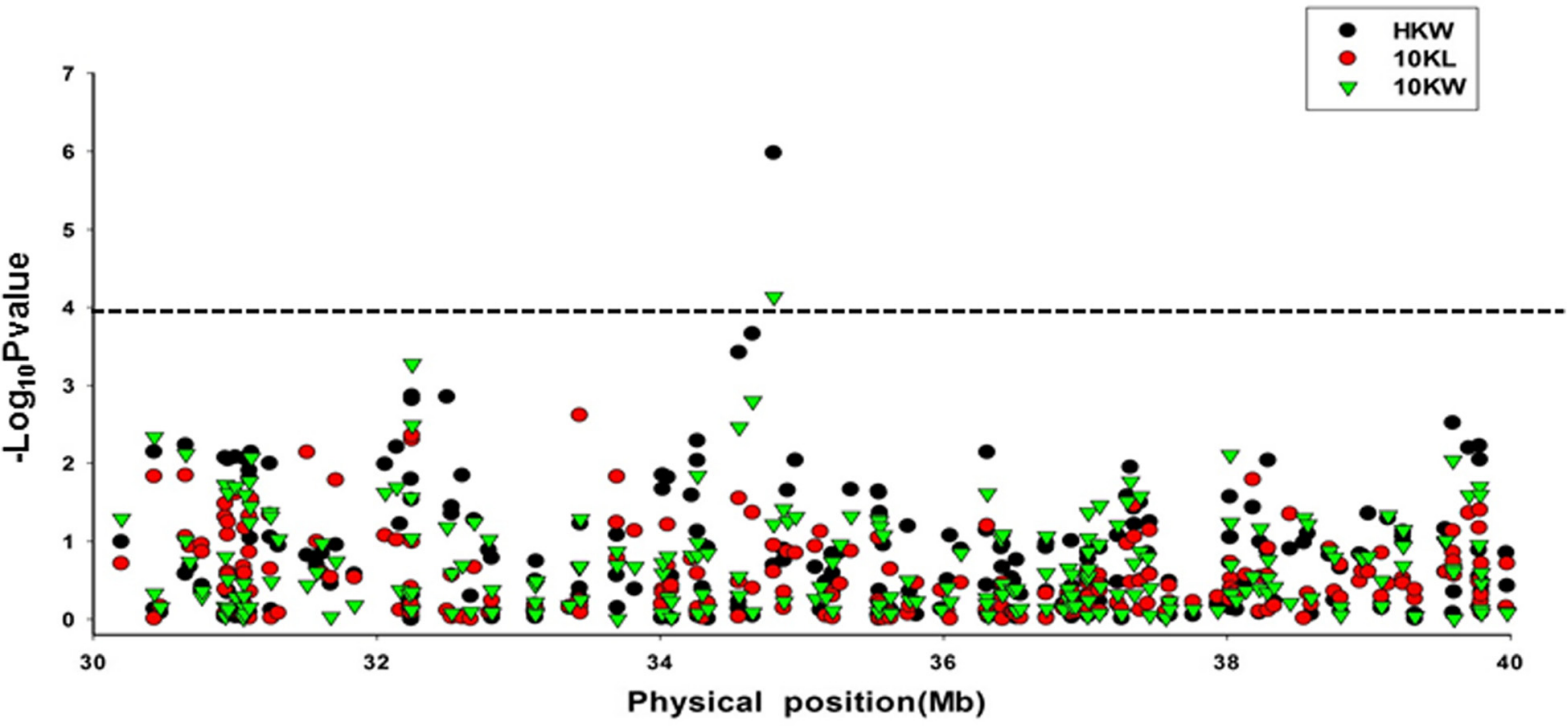
Results of regional association mapping. MLM tests at the region 30–40 Mb of chromosome 4. Only SYN4401 was significantly associated with 100-kernel weight and 10-kernel width (LOD>4)

We used the strategy of regional association mapping to further narrow down *qGW4.05* and identify candidate genes. An association mapping panel that contains 541 inbreed lines was field evaluated at three locations in 2 years. We selected single-nucleotide polymorphisms (SNP) markers in an interval (30–40 Mb) containing the sequence of UMC2061-BNLG1217 on chr4 of the maize genome. Using the mixed linear model, we identified one SNP, SYN4401, that was associated with the variation in kernel weight and 10-kernel width and explained 6.31 and 4.76 % of the phenotypic variation in kernel weight and kernel width, respectively (Fig. 5). However, no marker was identified that was significantly associated with kernel length.

### Prediction of candidate genes

The NO4-ND4M26 interval on the B73 genome is 279.6 kb long and contains only two genes (GRMZM2G702403 and GRMZM2G039934) and some transposable elements annotated in B73 reference genome v2.0 assembly (B73 RefGen_v2). Previous studies have demonstrated that GRMZM2G702403 is not expressed in developing kernels [35, 36]. The SNP SYN4401, which was identified by regional association mapping, is located in the gene GRMZM2G039934. We therefore considered this gene a candidate gene controlling kernel weight and size. GRMZM2G039934 encodes a putative leucine-rich repeat receptor-like protein kinase family protein. Sequencing revealed 18 SNPs and one Indel in the exons of this gene between HZS and LV28. These variations in the coding region cause eight amino acid substitutions (Table 3). SIFT analysis, which assesses whether an amino acid substitution affects the structure of a protein or its function, revealed that one of the eight substitutions was predicted with high confidence to result in the loss of protein function of GRMZM2G039934 (Table 3). The threonine encoded by the HZS allele is hydrophilic, whereas the isoleucine encoded by the LV28 allele is hydrophobic. This amino acid substitution may result in different protein functions that underlie the differences in 100-kernel weight and kernel size between HZS and LV28.

**Table 3:** Polymorphic sites causing amino acid changes in the protein of GRMZM2G039934

Gene ID	Amino acid substitution (Huangzaosi/LV28)^a^	PROVEAN score^b^	Prediction (cutoff = −2.5)^c^
GRMZM2G039934	R42C	−0.43	Neutral
	T55I	−3.72	Deleterious
	Q371R	−0.82	Neutral
	I375L	0.16	Neutral
	M380V	0.26	Neutral
	N386K	−0.09	Neutral
	K387I	−1.16	Neutral
	D388N	−0.65	Neutral

Notes: ^a^Amino acid substitution format is X#Y, where X is the original amino acid, # is the position of the substitution, and Y is the new amino acid. ^b^A delta alignment score is computed for each supporting sequence. The scores are then averaged within and across clusters to generate the final PROVEAN score. If the PROVEAN score is equal to or below a predefined threshold (e.g., −2.5), the protein variant is predicted to have a “deleterious” effect. If the PROVEAN score is above the threshold, the variant is predicted to have a “neutral” effect; ^c^for maximum separation of the deleterious and neutral protein variants, the default score threshold is currently set at −2.5 for binary classification

### Association mapping of the candidate gene and haplotype analysis

**Fig. 6:**
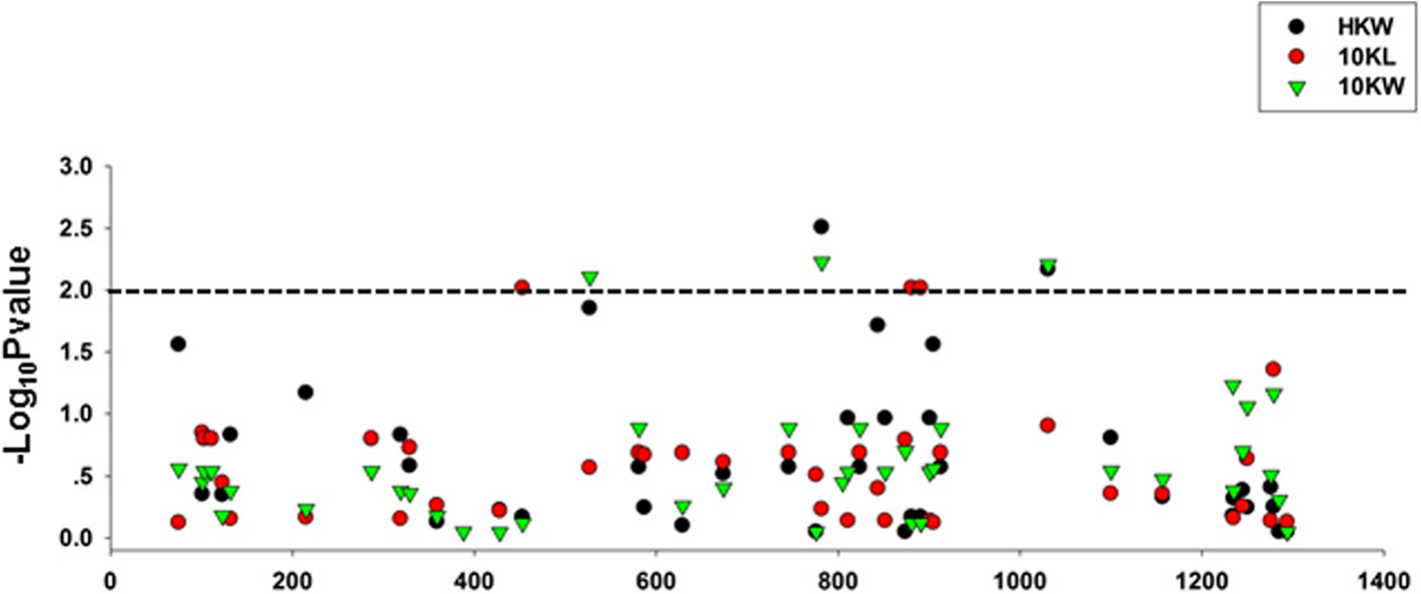
Association between the polymorphisms in *GRMZM2G039934* and HKW, 10KL and 10KW. All polymorphic sites with MAF ≥0.05 were used. The y axis represents the LOD score obtained by MLM on the panel of 184 inbred lines with variable kernel weights and kernel sizes. Six polymorphic sites in the gene *GRMZM2G039934* were significantly associated with kernel weight and kernel size

To determine the sites responsible for the differences in kernel size and kernel weight between HZS and LV28, the allelic variations of 19 sequence polymorphisms (Additional file 5: Figure S3) identified in HZS and LV28 were exclusively analysed in 184 inbred maize lines. The alleles in each polymorphic site with minor allele frequency >0.05 were used for association mapping using the mixed linear model (MLM), controlling for population structure (Q) and kinship (K) (MLM Q+K). The results revealed that one polymorphism (S453) in the coding region and two polymorphisms (S881and S891) in the intron were associated with kernel length, three polymorphisms (S527, S782 and S1031) in the coding region were associated with kernel width, and two polymorphisms (S782 and S1031) in the coding region were associated with kernel weight at the *P* < 0.01 level (Fig. 6). However, none of these polymorphisms generates an amino acid substitution.

**Fig. 7:**
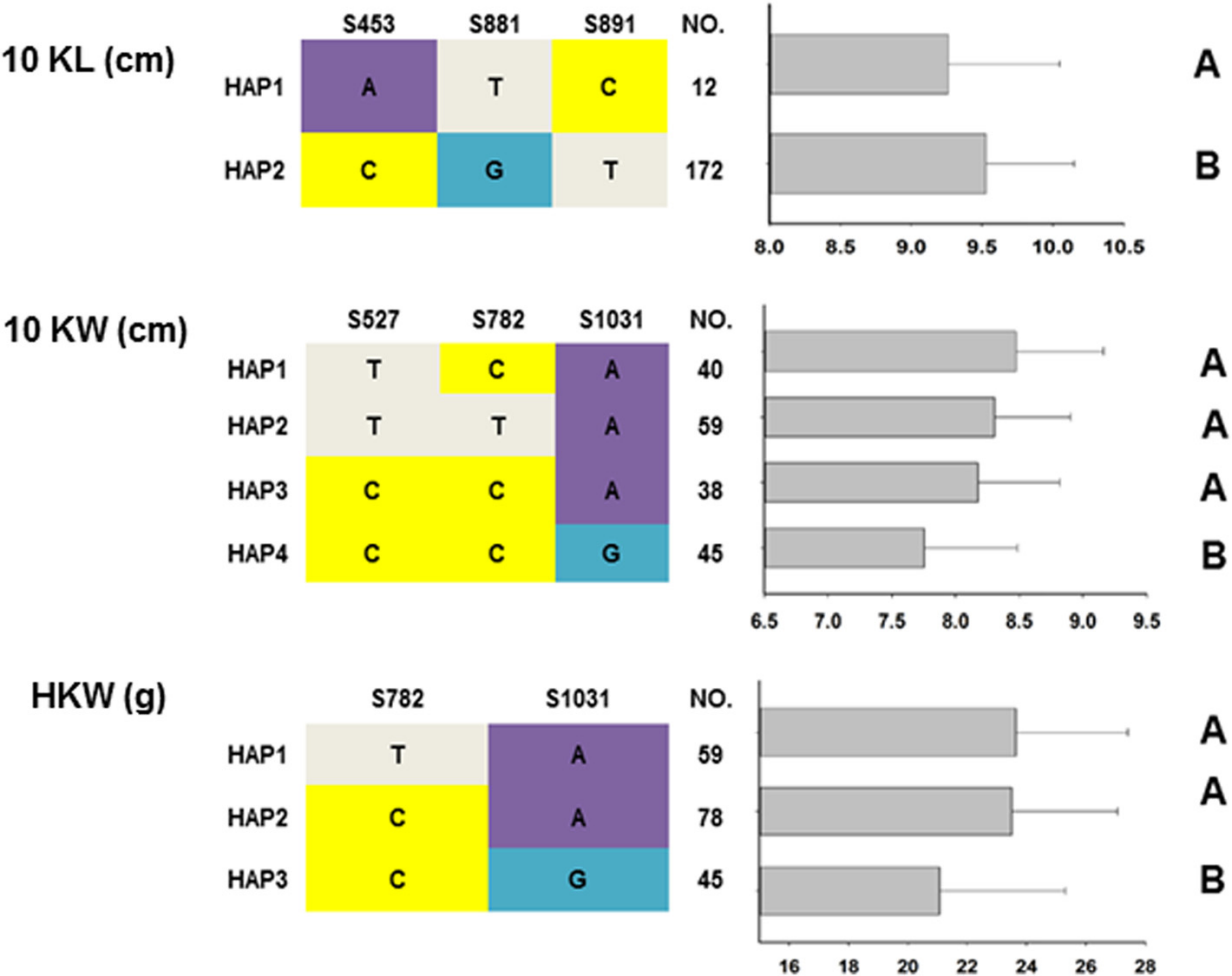
Phenotypic comparisons of different haplotypes for different traits. Different *letters* indicate statistically significant differences (*P* < 0.05), according to a pairwise *t* test. Haplotype analysis suggested that S453, S881 and S891, which associated with kernel length, might classify the population into two types. The two haplotypes differed significantly in kernel length at the *P* < 0.05 level. S527, S782 and S1031, which significantly associated with kernel width, could divide the panel into four haplotypes. The kernel width for haplotype 1, which corresponded to the HZS genotype, was significantly greater than that of haplotype 4, which corresponded to the LV28 genotype. S782 and S1031, which were related to 100-kernel weight, formed three different haplotypes. The phenotype of haplotype 3, which corresponded to the LV28 genotype, had a lower kernel weight than haplotype 2, which corresponded to the HZS genotype

Haplotype analysis suggested that S453, S881 and S891, which are associated with kernel length, might classify the population into two types. The two haplotypes differed significantly in kernel length at the *P* < 0.05 level (Fig. 7), but both the HZS and LV28 alleles belong to haplotype 2. S527, S782 and S1031, which are significantly associated with kernel width, may divide the panel into four haplotypes. The phenotypes of haplotype 1, haplotype 2 and haplotype 3 did not differ significantly but were significantly wider than haplotype 4 (Fig. 7). The kernel width for haplotype 1, which corresponds to the HZS genotype, was significantly higher than that of haplotype 4, which corresponds to the LV28 genotype, consistent with the kernel width difference between HZS and LV28. S782 and S1031, which are related to 100-kernel weight, form three different haplotypes (Fig. 7). The phenotype of haplotype 3, which corresponds to the LV28 genotype, had a smaller kernel weight than those of haplotypes 1, and haplotype 2 which corresponds to the HZS genotype.

## Discussion

### Comparison of *qGW4.05* and other major QTL for kernel weight and size

Kernel weight and size, as yield components, are typical quantitative traits that are controlled by multiple genes and sensitive to environmental impacts. The development of molecular markers has led to the identification of 200 QTL related to kernel weight and size distributed in the entire genome according to data in the MaizeGDB (http://www.maizegdb.org). In bin4.05, multiple QTL associated with yield components have been found: *qcobd8* for cob diameter [37], *qgyld12* for grain yield [31], *qkrow7* for kernel row number [37] and *qkw24* for kernel weight [30]. Peng et al. (2011) identified a QTL cluster for kernel weight and kernel length in bin4.05 with two F_2:3_ populations [38]. Li et al. (2011) and Wang et al. (2013) both identified a metaQTL associated with yield components by meta-analysis in bin4.05 [39, 40]. These results implied that *qGW4.05* with these QTL formed a core cluster for QTL controlling different kernel related traits.

Prado et al. (2014) have found multiple QTL related to kernel weight, located in bins 1.01, 1.05, 1.11, 3.06, 5.05, 9.05 and 10.03 [41]. Liu et al. (2014) identified 6, 16 and 15 QTL related to kernel length, kernel width and kernel weight, respectively [16]. Zhang et al. (2014) found 42 main-effect QTL related kernel weight and size [14]. Only a few of these QTL can be found in different genetic background and different environments. Among these QTL, digenic interactions involving multiple loci over the whole genome have been shown to be related to kernel weight and size. Like these QTL, *qGW4.05* can explain 23.94, 21.39 and 10.82 % of the phenotypic variance in hundred-kernel weight, 10-kernel length and 10-kernel width, respectively. Compared with the above QTL, *qGW4.05* can be found in many different populations including the F_2_ populations from the cross of maize and teosinte [30], the F_2_ population from a cross of B73 and A7 [31], the F_2:3_ populations from Huangzaosi and Qi319, the RIL population from Huangzaosi and other inbred lines [33, 38]. Based on the genetic linkage map constructed using 2091 bins as markers, we don’t found the digenic interaction between *qGW4.05* and other quantitative trait loci (data unpublished). These results suggested that the genetic bases of kernel weight and size are very complex and that positional cloning of these QTL will be very difficult. Compared with these QTL, *qGW4.05* may allow more efficient positional cloning of the candidate gene.

### *qGW4.05* is an important and pleiotropic locus

High-throughput SNP genotyping analysis of elite maize germplasm in China identified bin 4.05 as one of the conserved regions transmitted from Huangzaosi, an important foundation parent, to its descendants [42]. The locus *qGW4.05* is present across multiple environments and different genetic backgrounds such as Huangyesi3, LV28, QI319, Huobai and Duo229. Among the different populations, *qGW4.05* is related to multiple kernel traits. In the above populations, *qGW4.05*-HZS is positive for kernel-related traits, whereas other parents are negative for these traits. These results suggest that *qGW4.05* is very important for HZS and HZS-derived lines and is a positive QTL for kernel-related traits.

Many previous studies have indicated that yield and kernel-related traits are controlled by a set of QTLs, some of which are QTL clusters [9, 17, 18, 30, 32, 33, 38, 43–47]. The distribution of these QTL clusters can be explained by a pleiotropic QTL or multiple tightly linked QTLs. When a high-resolution map has been constructed, a QTL cluster can be resolved into many minor effect QTLs. QTL analysis in maize has clearly demonstrated that many complex traits controlled by QTL clusters, such as the grain yield, kernel size and other agronomic traits, can be broken down into many QTLs once the linkage map has been improved [33, 48, 49]. However, a QTL cluster may contain only one major QTL that controls multiple related traits and thus has pleiotropic effects. In the present study, QTL mapping in the RIL families restricted *qGW4.05* to a 10-Mb interval and revealed its relationship to both kernel size and kernel weight. When the interval was further narrowed to 1 Mb, *qGW4.05* remained associated with the three traits. This finding suggests that *qGW4.05* may be a pleiotropic locus that affects kernel size and kernel weight in maize.

### *GRMZM2G039934* is involved in the development of maize kernels via a different mechanism than in rice

In this study, we successfully fine-mapped *qGW4.05* to a 297.2 kb interval. Previous studies have indicated that only GRMZM2G039934 is expressed in this interval in kernels of maize [18, 19]. Regional association mapping revealed that the SNP SYN4401, which is located in GRMZM2G039934, is significantly associated with 100-kernel weight and 10-kernel width. We therefore propose that GRMZM2G039934 is a candidate gene related to the development of maize kernels. In rice, a 1-bp deletion in *GW2* results in a premature stop codon. The loss of function of *GW2* leads to an increased cell number, a wider spikelet hull and an accelerated grain milk-filling rate, which increases grain width, weight and yield [50]. Like *GW2*, a single SNP in exon2 of *GS3* results in a premature stop codon. The shorter protein is associated with a longer grain length and larger grain weight [44]. A 1212-bp deletion in *GW5* is associated with increased grain width in rice [45]. However, we did not identify any deletion or SNP changes resulting in a premature stop codon in GRMZM2G039934 in maize. Thus, the mechanisms underlying kernel development and regulation may differ between maize and rice.

GRMZM2G039934 encodes a putative leucine-rich repeat receptor-like protein kinase family protein. The protein product of the candidate gene is in the same family as *dwarf61*, which is involved in the brassinosteroid (BR) biosynthesis network and influences grain size development in rice [51]. Studies in *Arabidopsis* and rice have demonstrated that brassinosteroids play an important role in seed development [51–56]. Many BR-deficient mutants of *Arabidopsis* (*dwf5*, *shk1-D*) and rice (*brd2*, *dwf11*, *d61*) have a common phenotype that includes dwarfism, short organs, and small grains. Moreover, overexpression of BR biosynthesis-related genes increases grain size and the number of grains. These results suggest that BRs play a key role in normal seed development. However, the detailed mechanisms of BR regulation of seed development remain unclear. The rice dwarf mutant *d61* has a phenotype of smaller grains and lower kernel weight compared to wild type due to loss of function of the rice *brassinosteroid insensitive1* orthologue *OsBRI1* [51]. The mutants have higher biomass than wild type under high planting density. Moreover, the partial suppression of *OsBRI1* can increase grain yield by regulating the brassinosteroid biosynthesis network in transgenic rice plants. GRMZM2G039934 may be involved in the same biosynthetic process in maize*.* Detailed studies are necessary to reveal the mechanisms by which GRMZM2G039934 regulates kernel development in maize.

### *qGW4.05* for maize breeding

Maize is the most widely grown crop in the world, and to improve the grain yield has always been a top priority [57]. Identifying useful QTLs related to grain yield such as kernel weight, kernel size and kernel number is important for genetic manipulation to increase production via maize breeding. There are many successful examples of the introduction of useful QTLs. For example, the introduction of *qHSR1,* which is a QTL related to head smut in head smut–susceptible lines via marker-assisted selection, has significantly reduce disease incidence over time in maize [58, 59]. *qGW4.05* has been identified in different populations and in different environments [33]. In this study, the presence of *qGW4.05* was confirmed using two F_2_ populations of various sizes and regional association mapping analysis in a panel of 541 inbreed lines. Therefore, *qGW4.05* may be utilized in maize breeding by marker-assisted selection. The LV28 allele at *qGW4.05* decreases 100-kernel weight and kernel size relative to the HZS allele; thus, it may be feasible to use lines carrying the HZS allele to improve lines carrying the LV28 allele in *qGW4.05*. In particular, the two SNP sites S782 and S1031, which are associated with kernel weight and kernel width, could help breeders to select wider and heavier kernels of maize in the future.

## Conclusions

We combined linkage analysis and association mapping to fine-map and identify candidate gene(s) at *qGW4.05*, a major quantitative trait locus (QTL) associated with maize kernel weight and size. QTL *qGW4.05* was fine-mapped to a 279.6-kb interval in a segregating population derived from a cross of Huangzaosi with LV28. We identified GRMZM2G039934 as the candidate gene responsible for *qGW4.05*. Furthermore, six polymorphic sites in the gene GRMZM2G039934 were significantly associated with kernel weight and size. These results will improve our understanding of the genetic architecture and molecular mechanisms underlying kernel development in maize, which are important components of grain yield.

## Methods

### Plant materials used for fine-mapping of *qGW4.05*

*qGW4.05* controlling 100-kernel weight and kernel size was previously mapped to bin 4.05 of chromosome 4 using the RIL population from the cross of HZS and LV28 [33]. In the present study, we used G184, an RIL family from the above cross that harbours the LV28 allele of *qGW4.05*, to develop RIL-F_2_ with HZS. A total of 1332 RIL-F_2_ individuals were used to confirm the accurate physical location of *qGW4.05*. We then selected heterozygous individuals using markers flanking *qGW4.05* for self-pollination to develop the RIL-F_3_ population. The RIL-F_3_ population, which contained approximately 8000 individuals, was used to fine-map qGW4.05*.* Individuals containing recombination breakpoints within the QTL interval were selected from the RIL-F_3_ population for self-pollination to conduct a progeny test. Moreover, an association mapping panel (AP) with 541 inbred maize lines covering a wide range of genetic variation was used for regional association mapping. All plant materials in this study were conserved in our experiment lab and we declare that all plant materials in this study comply with the ‘Convention on the Trade in Endangered Species of Wild Fauna and Flora’.

### Field design and phenotypic evaluation

The RIL population was field evaluated previously [33]. The RIL-F_2_ and RIL-F_3_ populations were planted in summer 2012 and 2013 in Beijing (39.48° N, 116.28° E, in northern China). The progeny were tested in summer 2014 in Beijing. The association panel was field evaluated for the target phenotypes in nine environments: Changchun in Jilin province in 2011 (43.88° N, 125.35° E, in northeastern China), Beijing in 2011 and 2012, Tai’an in Shandong province in 2011 and 2012 (36.11° N, 117.08° E, in eastern China), Xinxiang in Henan province in 2011 and 2012 (30.77° N, 106.10° E, in central China), and Nanchong in Sichuan province in 2011 and 2012 (43.88° N, 125.35° E, in southwestern China). The institute of crop science belonging to the Chinese Academy of Agricultural Sciences has set up experimental field bases at all the above locations. The institute of crop science was approved for field experiments, and the field studies did not involve endangered or protected species.

The field experiment methodology and the evaluation of kernel-related traits for the populations used in this study were identical to those described in a previous study [33]. The populations were arranged in a randomized complete block design, and each genotype was grown in a single row 3 m in length with 0.6 m between adjacent rows, with 12 individual plants per row. The field management followed normal agricultural practices. After harvest, the kernels were threshed from the middle part of the ears to determine the 100-kernel weight (HKW, g), 10-kernel width (10KW, cm) and 10-kernel length (10KL, cm), which were estimated from the average of three measurements.

### Molecular marker development

The SSRs used for the RIL population were selected from MaizeGDB (http://www.maizegdb.org). According to re-sequencing information regarding HZS and LV28 provided by Professor Jinsheng Lai of China Agricultural University [60], PCR-based Indel markers and sequence-based SNP markers in the interval of the *qGW4.05* region were designed using Primer Premier 5.0 (PREMIER Biosoft International, USA) with a product size <300 bp. All markers are listed in Table 1 and were used to identify the genotype of the RIL-F_2_ and RIL-F_3_ populations. Of 56,110 SNPs derived from the MaizeSNP50 BeadChip within the confidence interval of *qGW4.05*, 256 SNPs were selected for association analysis of the association mapping panel (AP).

### Genotyping and QTL analysis

Genomic DNA was extracted from fresh maize seedling leaves using the cetyltrimethylammonium bromide (CTAB) method [61]. A marker linkage map was constructed using the Kosambi function of MAPMAKER/EXP version 3.0 [62]. A mixed model based on the composite interval mapping method was used to conduct QTL analysis by QTL IciMapping V3.3 [63, 64]. The threshold for indicating the existence of a significant QTL for 100-kernel weight and kernel size in each generation was obtained by 1000 permutations at a significance level of *P* = 0.05. The significance of the phenotypic differences for different recombinant types relative to LV28 or heterozygosis was evaluated using Student’s *t* test in SAS (SAS Institute, Inc., Cary, NC).

### Regional association mapping

Both the kinship matrix and the principal component analysis (PCA) were calculated using allelic data from 4544 SNP markers of 56,110 derived from the MaizeSNP50 BeadChip that were evenly distributed across the whole maize genome. Alleles of each polymorphism with minor frequency >0.05 were used for association mapping using the mixed linear model (MLM) controlling for population structure (Q) and kinship (K) (MLM Q+K). Significant marker-trait associations were declared for LOD>4. All associations were analysed with TASSEL5.0 [65, 66]. LD analysis within the target region was performed using the software Haploview [67].

### Candidate gene sequencing and association mapping

The genomic DNA sequences of candidate genes from HZS and LV28 were obtained by polymerase chain reaction (PCR) amplification using the primers N37F and N37R. PCR was performed using high-fidelity LA Taq Mix (Takara, http://www.clontech.com/takara). The purified PCR products were cloned into pLB-Vector (TIANGEN, http://www.tiangen.com) according to the manufacturer’s instructions. Three positive clones were sequenced for each sample. Sequence contig assembly and alignment were performed using DNAMAN version 5.2.2 (LynnonBiosoft, http://www.lynnon.com).

A subset of 184 inbred lines from the regional association mapping panel were used for candidate gene-based association mapping. The primers N37F/R were used to amplify the candidate gene’s coding region. The PCR products of three repetitions were directly sequenced. Initial alignment and manual refinement of the alignment were performed using BioEdit software [68]. Sites with allelic frequency >0.05 were used for subsequent analysis. Association mapping was performed with TASSEL 2.1 using an MLM Q+K model [65, 66].

### Ethics

The experiments comply with the ethical standards in the country in which they were performed.

### Consent to publish

Not applicable.

### Availability of data and materials

The data supporting the results of this article are included within the article and its additional files. The candidate gene (GRMZM2G039934) sequences of Huangzaosi and LV28 were deposited in the Genbank (https://www.ncbi.nlm.nih.gov/genbank) under accession number KU933938 and KU933939, respectively.

## Additional files

Additional file 1: Table S1. The primers used in this study for fine-mapping *qGW4.05* and candidate gene sequencing. (XLS 30 kb) Additional file 2: Table S2.
*qGW4.05* location in the RIL-F2 population in 2012 using the SSR markers. (DOCX 16 kb) Additional file 3: Figure S1. Validation of *qGW4.05* for 10KW in the RIL population in six different environments. The RILs were genotyped by using the markers NO4 and ND4M26. The distributions and mean values for 10KW are shown for the two homozygous genotypes, Huangzaosi and LV28, at the six experimental sites. Across the six environments, the RIL families that had the Huangzaosi homozygous genotype at the *qGW4.05* region had significantly wider kernels (*P* < 0.01) than the families that had the LV28 homozygous genotype. (TIF 112 kb) Additional file 4: Figure S2. Validation of *qGW4.05* for 10KL in the RIL population in six different environments. The RILs were genotyped by using the markers NO4 and ND4M26. The distributions and mean values for 10KL are shown for the two homozygous genotypes, Huangzaosi and LV28, at six experimental sites. Across all of the environments except Xinjiang-2010, the RIL families harbouring the *qGW4.05-Huangzaosi* allele had significantly longer kernels (*P* < 0.01) than the families harbouring the *qGW4.05-LV28* allele. (TIF 99 kb) Additional file 5: Figure S3. DNA sequence alignment of *GRMZM2G039934* between Huangzaosi and LV28. There were total 19 sequence polymorphisms between Huangzaosi and LV28, among which 17 sequence polymorphisms were located in the coding region; the others are located in the introns. (TIF 218 kb)

## Abbreviations

10KL: 10-kernel length
10-KW: 10-kernel width
AP: association mapping panel
B73 RefGen_v2: B73 reference genome v2.0 assembly
BR: brassinosteroid
HKW: hundred kernel weight
HZS: Huangzaosi
InDel: insert and deletion
K: kinship
MLM: mixed linear model
PCA: principal component analysis
PCR: polymerase chain reaction
Q: population structure
QTL: quantitative trait loci
RIL: recombinant inbred line
SNP: single-nucleotide polymorphisms
SSR: simple sequence repeat
