# Supplementary material for: Fine-mapping of qGW4.05, a major QTL for kernel weight and size in maize
Source: BMC Plant Biol. 2016 Apr 12;16:81. doi: 10.1186/s12870-016-0768-6 (PMC4828868; doi:10.1186/s12870-016-0768-6)
Supplement: Additional file 2: Table S2. — qGW4.05 location in the RIL-F2 population in 2012 using the SSR markers. (DOCX 16 kb) [file 12870_2016_768_MOESM2_ESM.docx]

**Table S2. *qGW4.05* location in the RIL-F2 population in 2012 using the SSR markers.**

| **Trait** | **Marker interval**^a^ | **LOD**^b^ | **PVE (%)**^c^ | **Add**^d^ | **Dom**^e^ |
| --- | --- | --- | --- | --- | --- |
| 10KL | UMC2061-BNLG1217 | 13.4895 | 5.1679 | -0.0225 | 0.0081 |
| 10KW | UMC2061-BNLG1217 | 7.8458 | 3.0149 | -0.0137 | 0.0047 |
| HKW | UMC2061-BNLG1217 | 7.7933 | 2.9767 | -0.9971 | 0.1732 |

Notes: Marker interval^a^, the flanking marker interval of the QTL; LOD^b^, logarithm of odds for each QTL; PVE (%)^c^, percentage of phenotypic variance explained by a QTL; A^d^, additive values (a positive value indicates that the additive effect was derived from LV28, and a negative value indicates derivation from Huangzaosi); D^e^, dominant values.
