# Supplementary figures and images for: Fine-mapping of qGW4.05, a major QTL for kernel weight and size in maize
Source: BMC Plant Biol. 2016 Apr 12;16:81. doi: 10.1186/s12870-016-0768-6 (PMC4828868; doi:10.1186/s12870-016-0768-6)

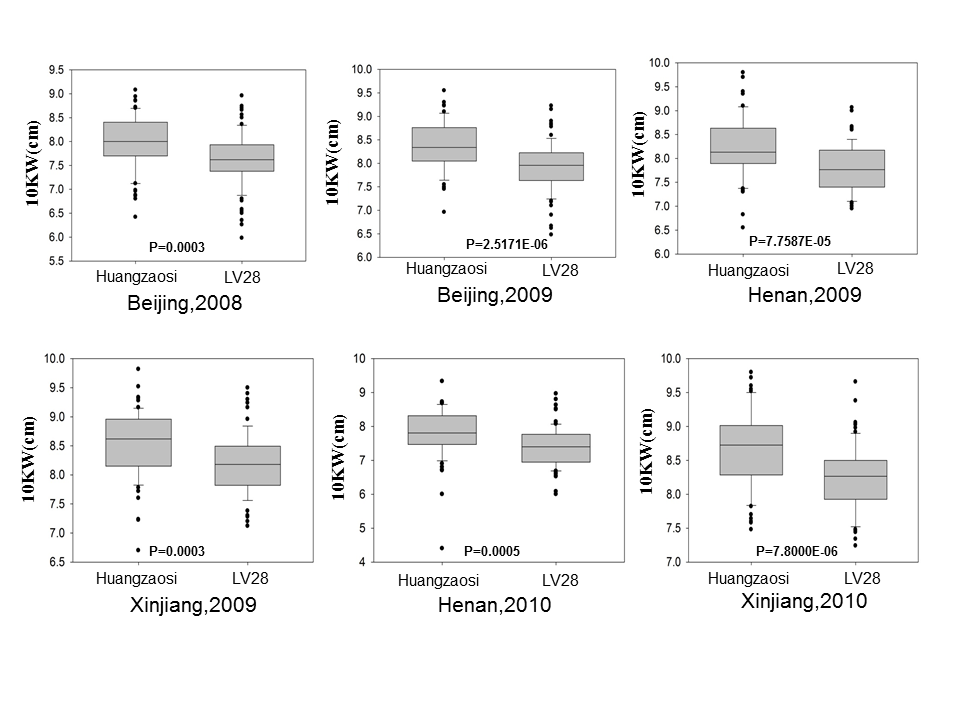

Supplement: Additional file 3: Figure S1. — Validation of qGW4.05 for 10KW in the RIL population in six different environments. The RILs were genotyped by using the markers NO4 and ND4M26. The distributions and mean values for 10KW are shown for the two homozygous genotypes, Huangzaosi and LV28, at the six experimental sites. Across the six environments, the RIL families that had the Huangzaosi homozygous genotype at the qGW4.05 region had significantly wider kernels (P < 0.01) than the families that had the LV28 homozygous genotype. (TIF 112 kb) [file 12870_2016_768_MOESM3_ESM.tif]

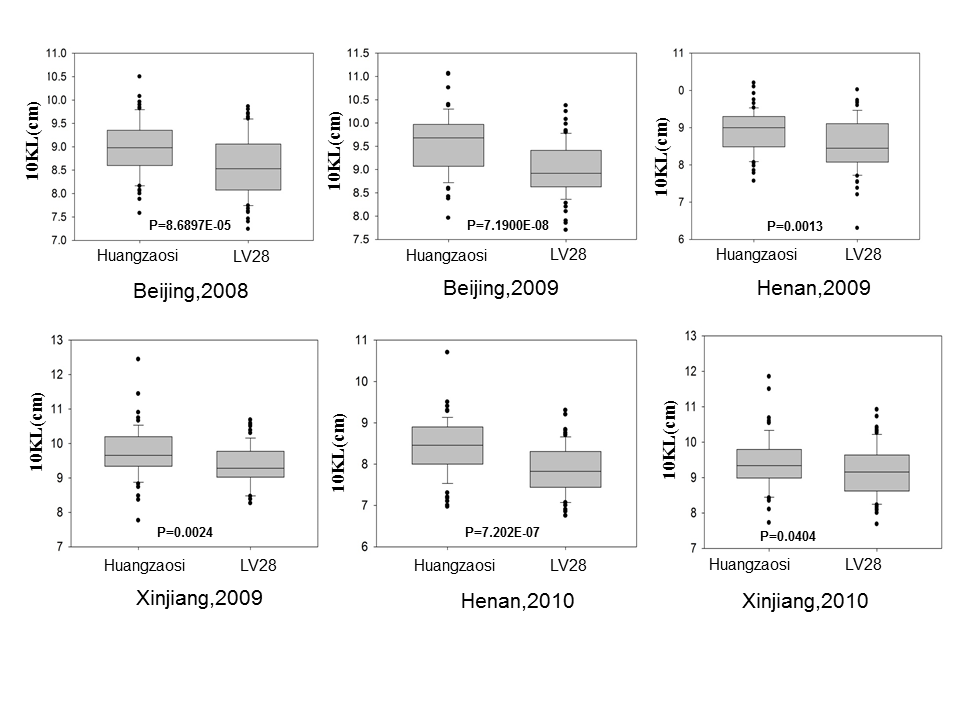

Supplement: Additional file 4: Figure S2. — Validation of qGW4.05 for 10KL in the RIL population in six different environments. The RILs were genotyped by using the markers NO4 and ND4M26. The distributions and mean values for 10KL are shown for the two homozygous genotypes, Huangzaosi and LV28, at six experimental sites. Across all of the environments except Xinjiang-2010, the RIL families harbouring the qGW4.05-Huangzaosi allele had significantly longer kernels (P < 0.01) than the families harbouring the qGW4.05-LV28 allele. (TIF 99 kb) [file 12870_2016_768_MOESM4_ESM.tif]

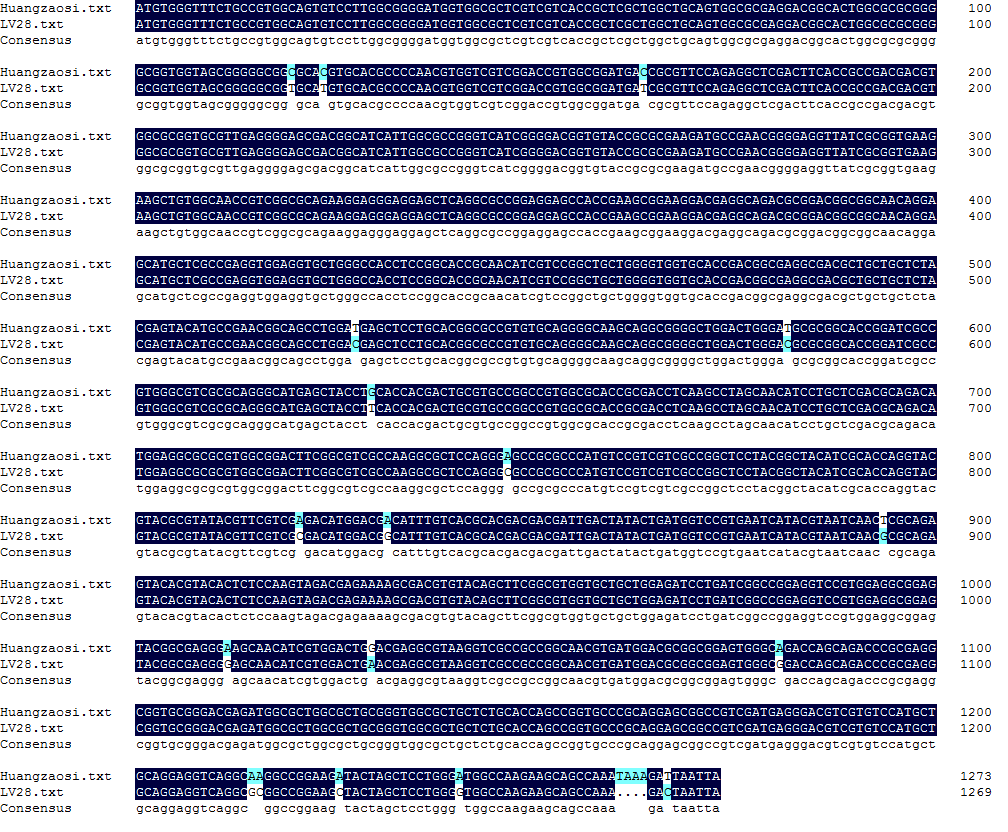

Supplement: Additional file 5: Figure S3. — DNA sequence alignment of GRMZM2G039934 between Huangzaosi and LV28. There were total 19 sequence polymorphisms between Huangzaosi and LV28, among which 17 sequence polymorphisms were located in the coding region; the others are located in the introns. (TIF 218 kb) [file 12870_2016_768_MOESM5_ESM.tif]
